# Supplementary material for: DNA-Based Method for Traceability and Authentication of Apis cerana and A. dorsata Honey (Hymenoptera: Apidae), Using the NADH dehydrogenase 2 Gene
Source: Foods. 2022 Mar 23;11(7):928. doi: 10.3390/foods11070928 (PMC8997381; doi:10.3390/foods11070928)
Supplement: Supplementary file 1 [file foods-11-00928-s001.zip › foods-1638960-supplementary.pdf]

Table S1. The honey samples, and their labels and localities used for analysis.

No.	*Entomological label*	*Locality*	*Co-ordination*	*Name*	*Provider*
1	*A. cerana*	Thailand, Chiang Mai	18.94°N, 99.36°E	CThai1	Beekeeper
2	*A. cerana*	Thailand, Chantaburi	12.66°N, 101.92°E	CThai2	Beekeeper
3	*A. cerana*	Thailand, Phayao	19.33°N, 99.92°E	CThai3	Beekeeper
4	*A. cerana*	Thailand, Chiang Rai	19.68°N, 99.35°E	CThai4	Beekeeper
5	*A. cerana*	Thailand, Patthalung	7.80°N, 100.01°E	CThai5	Beekeeper
6	*A. cerana*	Nepal, Pokhara	28.20°N, 83.98°E	CNep1	Beekeeper
7	*A. cerana*	Nepal, Pokhara	28.20°N, 83.58°E	CNep2	Beekeeper
8	*A. cerana*	South Korea	36.40°N, 128.49°E	CSK1	Beekeeper
9	*A. cerana*	South Korea	36.40°N, 128.49°E	CSK2	Beekeeper
10	*A. cerana*	South Korea	36.32°N, 128.48°E	CSK3	Beekeeper
11	*A. dorsata*	Thailand, Nakorn Sawan1	15.59°N, 100.43°E	DThai1	Honey hunter
12	*A. dorsata*	Thailand, Nakorn Sawan2	15.59°N, 100.43°E	DThai2	Honey hunter
13	*A. dorsata*	Thailand, Phetehaburi	12.95°N, 99.66°E	DThai3	Honey hunter
14	*A. dorsata*	Thailand, Chiang Mai	18.73°N, 98.94°E	DThai4	Honey hunter
15	*A. dorsata*	Thailand, Tak	17.05°N, 99.12°E	DThai5	Honey hunter
16	*A. mellifera*	South Korea	36.40°N, 128.49°E	MSK1	Beekeeper
17	*A. mellifera*	South Korea	36.40°N, 128.49°E	MSK2	Beekeeper
18	*A. mellifera*	South Korea	35.31°N, 127.59°E	MSK3	Beekeeper
19	*A. mellifera*	South Korea	36.32°N, 128.48°E	MSK4	Beekeeper
20	*A. mellifera*	South Korea	36.32°N, 128.48°E	MSK5	Beekeeper

Table S2. Mitochondrial sequences of honeybees *(A. cerana, A. dorsata* and *A. mellifera*) used to design species-specific primers.

No.	Sample ID number	Locality	GenBank accession no.	Reference
1	*Apis cerana*	China	KM244704	[55]
2	*Apis cerana*	China, Yunnan	GQ162109	[55]
3	*Apis cerana*	South Korea	KX908206	[56]
4	*Apis cerana*	South Korea	AP018431	[48]
5	*Apis cerana*	Malaysia	AP018149	[51]
6	*Apis cerana*	Japan, Nagasaki, Tsushima Island	AP017985	[52]
7	*Apis cerana*	Taiwan, Taipei	AP017984	[52]
8	*Apis cerana*	China, Jiangsu	AP017983	[52]
9	*Apis cerana*	Japan, Amami Oshima Island	AP017941	[52]
10	*Apis cerana*	Japan, Kyoto	AP017314	[54]
11	*Apis dorsata*	Thailand	AP018369	[53]
12	*Apis dorsata*	Thailand, Chiang Rai	KX908207	[56]
13	*Apis dorsata*	China	KC294229	[57]
14	*Apis mellifera capensis*	South Africa	MG552681	[43]
15	*Apis mellifera capensis*	South Africa	MG552682	[43]
16	*Apis mellifera capensis*	South Africa	MG552683	[43]
17	*Apis mellifera capensis*	South Africa	MG552684	[43]
18	*Apis mellifera capensis*	South Africa	MG552685	[43]

19	*Apis mellifera capensis*	South Africa	MG552686	[43]
20	*Apis mellifera capensis*	South Africa	MG552687	[43]
21	*Apis mellifera capensis*	South Africa	MG552688	[43]
22	*Apis mellifera capensis*	South Africa	MG552689	[43]
23	*Apis mellifera capensis*	South Africa	MG552690	[43]
24	*Apis mellifera capensis*	South Africa	MG552691	[43]
25	*Apis mellifera capensis*	South Africa	MG552692	[43]
26	*Apis mellifera capensis*	South Africa	MG552693	[43]
27	*Apis mellifera capensis*	South Africa	MG552694	[43]
28	*Apis mellifera capensis*	South Africa	MG552695	[43]
29	*Apis mellifera capensis*	South Africa	MG552696	[43]
30	*Apis mellifera capensis* A. mellifera scutellata*	South Africa	MG552697	[43]
31	*Apis mellifera scutellata*	South Africa	MG552698	[43]
32	*Apis mellifera scutellata*	South Africa	MG552699	[43]
33	*Apis mellifera scutellata*	South Africa	MG552700	[43]
34	*Apis mellifera scutellata*	South Africa	MG552701	[43]
35	*Apis mellifera scutellata*	South Africa	MG552702	[43]
36	*Apis mellifera scutellata*	South Africa	MG552703	[43]
37	*Apis mellifera monticola*	east African honey bee, reared in Germany	MF678581	[40]
38	*Apis mellifera*	Jordan?	MF059100	Haddad, unpublished
39	*Apis mellifera mellifera*	Norway	KY926884	Eimanifar et al., unpublished
40	*Apis mellifera intermissa*	Morocco	KY926883	Eimanifar et al., unpublished
41	*Apis mellifera scutellata*	Germany	KY614238	Eimanifar et al., unpublished
42	*Apis mellifera lamarckii*	Germany (Egypt source)	KY464958	[41]
43	*Apis mellifera meda*	Germany	KY464957	[42]
44	*Apis mellifera ligustica*	Italy	KX908209	[56]
45	*Apis mellifera capensis*	South Africa	KX870183	[39]
46	*Apis mellifera syriaca*	Levant Region	KP163643	[46]
47	*Apis mellifera intermissa*	Algeria	KM458618	[47]
48	*Apis mellifera scutellata*	Mexico: Queretaro	KJ601784	[45]
49	*Apis mellifera mellifera*	?	KJ396181	[44]
50	*Apis mellifera*	New Zealand	AP018435	[49]
51	*Apis mellifera*	United Kingdom	AP018432	[50]

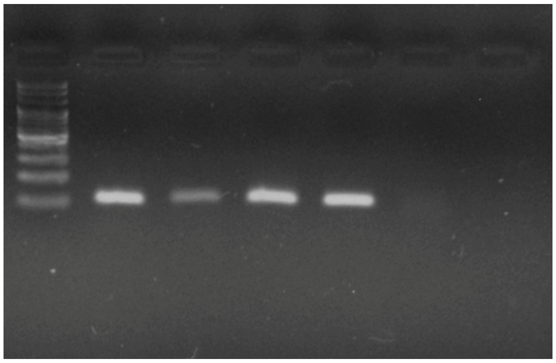

Figure S1. Preliminary specificity test of ACF/ACR (Suarez et al., 2018) using DNA extracts of honeybees. DNA: Lane 1, *A. dorsata* (Nepal); Lane 2, *A. dorsata* (Thailand); Lane 3, *A. cerana* (South Korea); Lane 4, *A. cerana* (Nepal); Lane 5, *A. mellifera* (South Korea); Lane 6, *A. mellifera* (Thailand); Lane M, DNA marker.

References

40. 39. Eimanifar, A., R. T. Kimball, E. L. Braun, and J. D. Ellis. 2016. The complete mitochondrial genome of the Cape honey bee Esch., Apis mellifera capensis (Insecta: Hymenoptera: Apidae). Mitochondrial DNA Part B 1: 817–819.

40. Eimanifar, A., R. T. Kimball, E. L. Braun, S. Fuchs, B. Grünewald, and J. D. Ellis. 2017a. The complete mitochondrial genome of an east African honey bee, Apis mellifera monticola Smith (Insecta: Hymenoptera: Apidae), Mitochondrial DNA Part B 2(2): 589–590.

41. Eimanifar, A., R. T. Kimball, E. L. Braun, D. M. Moustafa, N. Haddad, S. Fuchs, B. Grunewald, and J. D. Ellis. 2017b. The complete mitochondrial genome of the Egyptian honey bee, Apis mellifera lamarckii (Insecta: Hymenoptera: Apidae). Mitochondrial DNA Part B 2: 270–272.

42. Eimanifar, A., R. T. Kimball, E. L. Braun, S. Fuchs, B. Grunewald, and J. D. Ellis. 2017c. The complete mitochondrial genome of Apis mellifera meda (Insecta: Hymenoptera: Apidae). Mitochondrial DNA Part B 2: 268–269.

43. Eimanifar, A., R. T. Kimball, E. L. Braun, and J. D. Ellis. 2018. Mitochondrial genome diversity and population structure of two western honeybee subspecies in the Republic of South Africa. Sci. Rep. 8: 1333.

44. Fuller, Z. L., E. L. Nino, H. M. Patch, O. C. Bedoya-Reina, T. Baumgarten, E. Muli, F. Mumoki, A. Ratan, J. McGraw, M. Frazier, D. Masiga, S. Schuster, C. M. Grozinger, and M. Miller 2015. Genome-wide analysis of signatures of selection in populations of African honey bees (Apis mellifera) using new web-based tools. BMC Genomics 16: 518.

45. Gibson, J. D., and G. J. Hunt. 2016. The complete mitochondrial genome of the invasive Africanized honey bee, Apis mellifera scutellata (Insecta: Hymenoptera: Apidae). Mitochondrial DNA Part A 27(1): 561–562.

46. Haddad, N. J. 2016. Mitochondrial genome of the Levant Region honey bee, Apis mellifera syriaca (Hymenoptera: Apidae). Mitochondrial DNA Part A 27(6): 4067–4068.

47. Hu, P., Z. X. Lu, N. Haddad, A. Noureddine, W. Loucif-Ayad, Y. Z. Wang, R. B. Zhao. A. L. Zhang, X. Guan, H. X. Zhang, and H. Niu. 2016. Complete mitochondrial genome of the Algerian honey bee, Apis mellifera intermissa (Hymenoptera: Apidae). Mitochondrial DNA Part A 27(3): 1791–1792.

48. Ilyasov, R. A., J. Park, J. Takahashi, H. W. Kwon. 2018. Phylogenetic uniqueness of honeybee Apis cerana from the Korean peninsula inferred from the mitochondrial, nuclear, and morphological data. J. Apicul. Sci. 62 (2): 189–214.

49. Nakagawaa, I., M. Maedaa, M. Chikanoa, H. Okuyamaa, R. Murrayb, and J. Takahashia. 2018. The complete mitochondrial genome of the yellow coloured honeybee Apis mellifera (Insecta: Hymenoptera: Apidae) of New Zealand. Mitochondrial DNA Part B 3(1): 66–67.

50. Okuyama, h., J. Hill, S. J. Martin, and J. Takahashi. 2018. The complete mitochondrial genome of a Buckfast bee, Apis mellifera (Insecta: Hymenoptera: Apidae) in Northern Ireland. Mitochondrial DNA Part B 3(1): 338–339.

51. Okuyama, H., S. Tingek, and J. Takahashi. 2017a. The complete mitochondrial genome of the cavity-nesting honeybee, Apis cerana (Insecta: Hymenoptera: Apidae) from Borneo, Mitochondrial DNA Part B 2(2): 475–476.

52. Okuyama, H., R. Jimi, T. Wakamiya, and J. Takahashi. 2017b. Complete mitochondrial genome of the honeybee Apis cerana native to two remote islands in Japan. Conserv. Genet. Resour. 9: 557–560

53. Takahashi, J., S. Deowanish, and H. Okuyama. 2017. Analysis of the complete mitochondrial genome of the giant honeybee, Apis dorsata, (Hymenoptera: Apidae) in Thailand. Conserv. Genet. Resour. https://doi.org/10.1007/s12686-017-0942-7

54. Takahashi, J., T. Wakamiya, T. Kiyoshi, H. Uchiyama, S. Yajima, K. Kimura, and T. Nomura. 2016. The complete mitochondrial genome of the Japanese honeybee, Apis cerana japonica (Insecta: Hymenoptera: Apidae). Mitochondrial DNA Part B 1(1): 156–157.

55. Tan, H.W., G. H. Liu, X. Dong, R. Q. Lin, H. Q. Song, S. Y. Huang, Z. G. Yuan, and X. Q. Zhao. 2011. The complete mito-chondrial genome of the Asiatic cavity nesting honeybee Apis cerana (Hymenoptera: Apidae). PLoS ONE 6(8): e23008.

56. Wang, A. R., J. S. Kim, M. J. Kim, H. K. Kim, Y. S. Choi, and I. Kim. 2018. Comparative description of mitochondrial genomes of the honey bee Apis (Hymenoptera: Apidae): four new genome sequences and Apis phylogeny using whole genomes and individual genes. J. Apic. Res. 57(4): 484–503.

57. Yang, J., J. Xu, J. Wu, X. Zhang, and S. He. 2019. The complete mitogenome of wild honeybee Apis dorsata (Hymenoptera: Apidae) from South-Western China. Mitochondrial DNA Part B 4(1): 231–232.
